# Using a Multicomponent Implementation Strategy to Increase Adoption and Effectiveness of a Universal Mental Health Prevention Program in Australian Primary Schools: a Cluster Randomized Trial Using a Type-3 Hybrid Design

**DOI:** 10.1007/s11121-025-01870-3

**Published:** 2026-02-06

**Authors:** Rachel Baffsky, Quincy J.J. Wong, Patricia Cullen, Aliza Werner-Seidler, Alison L. Calear, Philip J. Batterham, John W. Toumbourou, Lauren McGillivray, Bridianne O’Dea, Rebecca Ivers, Michelle Torok

**Affiliations:** 1 Black Dog Institute, University of New South Wales, Sydney, NSW Australia; 2 School of Population Health, University of New South Wales, Sydney, NSW Australia; 3 School of Psychology, Western Sydney University, Sydney, NSW Australia; 4 Centre for Mental Health Research, Australian National University, Canberra, ACT Australia; 5 School of Psychology, SEED Lifespan Research Centre, Deakin University, Geelong, VIC Australia; 6 College of Education, Psychology and Social Work, Flinders University Institute for Mental Health and Wellbeing, Flinders University, Adelaide, SA Australia

**Keywords:** School, Suicide prevention, Mental health prevention, Good Behavior Game, Implementation, Effectiveness, Trial

## Abstract

It has been demonstrated in multiple randomized trials that the PAX Good Behavior Game is an effective school-based program for reducing the severity and progression of childhood emotional and behavioral problems that are prognostic of mental disorders, personality disorders, and self-harm behavior in adolescence and early adulthood. Embedding effective programs into routine practices of frontline settings is a global priority for mental health prevention, however, little is known about what strategies effectively support program implementation in schools. The purpose of this study was to test the effect of a multicomponent implementation strategy on the adoption and efficacy of the PAX Good Behavior Game, using a cluster randomized controlled trial with a type-3 implementation-effectiveness design. The trial was conducted in 25 primary schools across New South Wales, Australia. Schools were randomly assigned to receive the PAX Good Behavior Game *plus* an implementation toolkit to support adoption (intervention group) or the PAX Good Behavior Game only (control group). The primary outcome was change in rate of program adoption, measured as current use or support of the PAX Good Behavior Game, first measured at 6 weeks post-registration (T0). The secondary effectiveness measure was change in students’ emotional and behavioral problems, using the teacher-reported Strengths and Difficulties Questionnaire (SDQ). The first measure of the SDQ occurred at trial registration, prior to program implementation (T0). Both adoption and effectiveness were re-measured at 6 months post-registration (T1). All data were analyzed using intention-to-treat methods. Early program adoption was higher in the intervention group compared to control group (93.6% vs 45.2%: OR = 21.20, 95% CI [3.50, 128.45],* z* = 3.32, *p* < 0.001), with no differential effects at 6 months (96.8% vs 95.7%: OR = 1.21, 95% CI [0.16, 9.04], *z* = 0.18, *p* = 0.855). Emotional and behavioral problems reduced in both groups from baseline to 6 months (*p* < 0.001, *d* = − 0.25), with no differential effects observed at T1 (*p* = 0.474, *d* = − 0.08). There was a positive linear relationship between adoption and the perceived acceptability, appropriateness, and feasibility of the program for the pooled sample. It seems that providing implementation support early in the process of establishing new innovations in schools may help accelerate early adoption by increasing perceived appropriateness and acceptability, but uncertainty remains as to what support is needed to optimize implementation and effectiveness longer term. The protocol was retrospectively registered with the Australian New Zealand Clinical Trials Registry, ACTRN12621001125819.

**Supplementary Information:**

The online version contains supplementary material available at 10.1007/s11121-025-01870-3.

## Introduction

Mental disorders are the biggest contributor to disease burden in children and young people aged 5 to 24 years, accounting for 20% of years lived with disability (Kieling et al., 2024). Most common adult mental health problems have roots in early life (Copeland et al., 2015; Mulraney et al., 2021), and attenuating the potential lifetime illness and dysfunction requires effective preventative interventions to be delivered before risk can exert influence. Based on global estimates that 92% of children aged 4–12 years attend school (Roser, 2021), primary (or elementary) schools are an obvious setting for wide-reaching prevention efforts. A growing body of high-quality evidence shows that exposure to school-based mental health primary prevention programs improves mental health symptoms (Werner-Seidler et al., 2021; Zhang et al., 2023) and reduces the incidence of many externalizing disorders (Kellam et al., 2011). Although the effect sizes of such programs are small on average (*g* = 0.17–0.18) (Werner-Seidler et al., 2021; Zhang et al., 2023), their impacts may be profound if sufficient population exposure is achieved.

### The Good Behavior Game

The Good Behavior Game (and the commercially distributed PAX Good Behavior Game variant in particular) is one of the few primary prevention programs shown to improve the emotional and behavioral problems in childhood that are prodromal to common mental disorders and related behaviors, such as deliberate self-harm (Kellam et al., 2011). The program uses an integrated classroom delivery model, where micro-strategies are woven into the classroom operation and curricula to target transdiagnostic factors implicated in the escalation and maintenance of emotional and behavioral problems, e.g., self-regulation and relational behavior (Domitrovich et al., 2015). These risk factors are modified using operant conditioning mechanisms of behavioral monitoring: positive reinforcement and positive punishment (Johansson et al., 2020). An extensive body of experimental research on the Good Behavior Game shows that there are small, yet significant, crossover preventative effects on a range of behavioral outcomes, including aggression, attention deficit hyperactivity disorder, conduct disorder, and self-injurious thoughts and behaviors (i.e., Hedge’s *g* 0.10–0.49) (Smith et al., 2021). Yet, despite the clear benefits of this program, it is subject to implementation challenges that threaten how it is adopted and sustained in schools.

### Implementation Challenges

An increasing number of studies show teacher dissatisfaction related to implementing the Good Behavior Game. Most commonly, teachers report that the limited training and lack of refresher training affect the development of self-efficacy to deliver the program with fidelity, and that inadequate leadership and material support affect motivation to sustain delivery (Coombes et al., 2016; Domitrovich et al., 2015; Jack et al., 2020). A recent meta-analysis, including eight randomized controlled trials (RCTs) of the Good Behavior Game, found that these implementation barriers were significantly associated with variations in delivery fidelity and quality (Ulla et al., 2022). There is also evidence that the fidelity of implementing the Good Behavior Game is generally low, with only one trial demonstrating acceptable fidelity (> 80% components present) (Smith et al., 2021). Such findings suggest that in many cases, the program may not be achieving its intended benefits. As high-quality implementation can increase the magnitude of program effectiveness by 12-fold (Durlak & DuPre, 2008), overcoming such challenges with purpose-designed implementation strategies that address structural, motivational, and leadership barriers to delivery is an important imperative for optimizing mental health prevention in schools.

### Implementation Strategies

There is promising evidence that the use of certain implementation strategies, including monitoring and feedback (Wolfenden et al., 2017), leadership support (Dijkman et al., 2017; Wolfenden et al., 2019), and program champions (Nadeem et al., 2018), can optimize teachers’ motivation and self-efficacy to delivery of school-based health prevention programs. However, understanding where to focus implementation efforts is challenging, as there is much uncertainty in the literature as to what specific strategies drive intervention response, and whether a multifaceted approach is likely to produce greater effects relative to a single-component approach (Squire et al., 2014). In school-based research, pooled analyses indicate that multifaceted implementation strategies may be slightly more effective than single-component approaches (*g* = 2.60 vs *g* = 2.18) (Merle et al., 2022), while reviews in the broader health literature suggest no difference (Squire et al., 2014). There are methodological issues in these reviews, however, which may bias these findings and perpetuate uncertainty as to the better approach, including the use of lower quality, uncontrolled studies (Baffsky et al., 2023a; Merle et al., 2022), and lack of clarity about how strategies were designed and what they target (Squire et al., 2014). High-quality experimental trials to test the causal effects of strategies purpose designed to overcome setting-specific barriers to implementation are needed to refine this field.

To date, only one rigorous implementation science trial has been conducted using the Good Behavior Game (Merle et al., 2023), designed to address the challenge of low uptake of new practices following training. The study tested a theory-driven, adaptive training strategy, delivered prior to, and following, the standard Good Behavior Game training, targeting teachers’ attitudes, beliefs, and intentions towards the program. Teachers who were nonresponsive to the initial training received additional motivational interventions to increase the likelihood of adoption. The study showed that the adaptive training approach had an immediate positive impact on adoption, which was not sustained over time (Merle et al., 2023). These findings suggest that motivationally focused, pre-implementation strategies may have time-limited effects on program delivery, highlighting the need to consider, and test, a broader combination of short- and longer-term focused strategies to achieve and maintain adoption.

### Research Gap and Aims

Due to the low quality of implementation research and limited experimental trials involving the Good Behavior Game, there remains substantial uncertainty as to what strategies support effective uptake and implementation of the Good Behavior Game over time. Rigorous, theory-driven efforts to test implementation strategies that target factors related to early adoption and program longevity are needed if we are to advance understanding of how to support teachers to adopt school-based practices that can benefit the mental health and well-being of children and adolescents.

As stated in our protocol paper (Baffsky et al., 2022), the primary aim of this hybrid implementation-effectiveness RCT was to test the hypothesis that a multicomponent implementation strategy delivered alongside the PAX Good Behavior Game would lead to higher rates of program adoption (implementation outcome) compared to the PAX Good Behavior Game alone. Given the uncertainty about best implementation practice, we designed a multicomponent strategy to address common co–occurring barriers to adoption and adherence identified in the literature, including lack of leadership support, time for delivery, and training (Shoesmith et al., 2021). This study is the first randomized trial to test a strategy that targets multiple domains in which barriers to implementation are known to occur (Ulla et al., 2022). Our secondary aim was to examine whether students in the intervention group showed greater improvements in emotional and behavioral problems compared to students in the control group (effectiveness outcome), given that high-quality implementation is a known mediator of effectiveness (Durlak & DuPre, 2008). Secondary implementation outcomes relating to the acceptability, feasibility, and appropriateness of the PAX Good Behavior Game were examined for variance between groups, and as potential mediators of program adoption. As our implementation strategy was designed to overcome common barriers to the delivery of PAX Good Behavior Game, we hypothesize that there will be higher rates of perceived acceptability, feasibility, and appropriateness in the intervention group, resulting in a higher likelihood of adoption.

## Methods

This study was performed in line with the principles of the Declaration of Helsinki. The study was prospectively approved by the University of New South Wales Human Research Ethics Committee (No. HC200759, 4 Dec 2020) and the NSW Government State Education Research Applications Process (No. 2020364, 2 Dec 2020). The protocol was registered with the Australian New Zealand Clinical Trials Registry (ANZCTR) following ethics approval, ACTRN12621001125819. Unanticipated delays in ethics approvals and concurrent time constraints related to the trial funding meant that school recruitment had to commence in January 2021, resulting in retrospective registration with ANZCTR. Trial outcomes are reported as per protocol.

### Study Design and Participants

We conducted a two-arm parallel cluster RCT using a type-3 hybrid implementation-effectiveness design (Curran et al., 2012) in New South Wales primary schools (for students aged 4–12 years), Australia. At the school level, eligibility criteria were government (public) primary schools (covering kindergarten to year 6), located in New South Wales, and where there was at least one teacher enrolled in the Department of Education’s PAX Good Behavior Game virtual training at the time of study registration or who had undertaken the training within the past month. All study data was collected only from school staff, and eligible individuals were those in eligible schools who were either responsible for delivering the PAX Good Behavior Game in their classroom (teachers), or in a position of providing executive or administrative support for the implementation of the program (other school staff). There were no specific exclusion criteria.

### Power Calculation

We estimated that a total sample of 184 educators from 46 schools (92 per group; ~ 4 staff per school) would be required to detect an effect of *d* = 0.4 on program adoption and secondary implementation outcomes with 80% power, an intraclass correlation coefficient of 0.05, and an alpha of 0.05. This calculation assumes that 78% of intervention schools and 40% of control schools would be delivering the PAX Good Behavior Game at 6 months following trial registration (T1), using estimates from prior community-based research (Fagan et al., 2012). Accounting for 30% attrition between baseline (T0) and T1, our intended sample was 206 staff (*n* = 103 per group).

### Sample Characteristics

Although our recruitment period coincided with the COVID–19 pandemic, we enrolled 28 schools into the trial between 27 January 2021 and 21 February 2022. The final T1 assessment took place on August 27, 2022. Three schools withdrew from the study after school level allocation had been performed, due to COVID-related constraints (Fig. 1). Our final sample consisted of 187 educators from 25 schools (92% of target sample) (Table 1). Nine schools (36%) were located in metropolitan/urban areas (intervention: *n* = 6; control: *n* = 3; e.g., City of Sydney, Greater Western Sydney) and 16 (64%) in regional areas (intervention: *n* = 7, control: *n* = 9; e.g., Riverina, Northern Rivers, Illawarra and Greater Newcastle). Approximately half the schools were “small” (*n* = 13, 52%; intervention: *n* = 7, control: *n* = 6), with total student populations of < 300 (*M* student population 268.1, SD = 204.5). The mean Index of Community Socio-Educational Advantage score, a numerical scale used in Australia to represent the level of educational advantage or disadvantage that students bring to school based on their socio-economic backgrounds, was 952.0 (SD 83.5; range 781–1146). As the average ICSEA score is 1000, the schools in the study were representative of the Australian student population.

**Table 1:** Baseline sample characteristics

	Intervention (PAX GBG + PAX Plus)	Control (PAX GBG)	Total sample
Educators			
	*N* = 94	*N* = 93	*N* = 187
Age in years, M (SD)	38.6 (8.5)	40.4 (9.3)	39.4 (8.9)
Gender identity, *n* (%)			
Female	82 (87.2)	77 (82.8)	159 (85.0)
Male	11 (11. 7)	12 (12.9)	23 (12.3)
Non-binary	1 (1.1)	4 (4.3)	5 (2.7)
Total years’ experience as an educator n (%)*			
≤ 5	26 (27.7)	20 (21.5)	46 (24.6)
6–10	23 (24.5)	24 (25.8)	47 (25.1)
≥ 11	44 (46.8)	44 (47.3)	88 (47.1)
School size, *n* (%)			
< 300	74 (78.7)	25 (26.9)	99 (52.9)
≥ 300	20 (21.3)	68 (73.1)	88 (47.1)
School location, *n* (%)			
Metropolitan/urban	36 (38.3)	63 (67.7)	99 (52.9)
Regional	58 (61.7)	30 (32.3)	88 (47.1)
Students			
	*N* = 1445	*N* = 1553	*N* = 2998
Age in years, M (SD)	7.32 (1.88)	7.49 (1.53)	7.40 (1.71)
Biological sex, *n* (%)			
Male	761 (52.7)	813 (52.4)	1574 (52.5)
Female	684 (47.3)	740 (47.6)	1424 (47.5)
School grade, *n* (%)			
Kindergarten	346 (23.9)	267 (17.2)	613 (20.4)
Year 1	295 (20.4)	262 (16.9)	557 (18.6)
Year 2	283 (19.6)	265 (17.1)	548 (18.3)
Years 3–6	521 (36.1)	759 (48.9)	1280 (42.7)

*M*, mean; *SD*, standard deviation; *PAX GBG*, PAX Good Behavior Game

^#^Missing data for *n* = 4 control participants

^*^Missing data for *n* = 1 intervention participant and *n* = 5 control participants

Fig. 1 CONSORT Trial Flow 

### Randomization and Masking

Schools (clusters) were randomly allocated 1:1:1:1 in blocks of four to either receive the PAX Good Behavior Game plus a multi-strategy implementation toolkit specifically designed for this study, called “PAX Plus” (Intervention group), or the PAX Good Behavior Game only (Control group). Individual-level consent was obtained from staff subsequent to the random allocation process. Randomization was stratified by school size (≥ 300 students, < 300 students) and regionality (regional, urban). The randomization schedule was computer-generated using the Sealed Enveloped blocked randomization online tool (Sealed Envelope Ltd, 2022), and this was done by a statistician independent of the research team. A list of schools and their allocation outcome was provided by the statistician to the project research officer. Control schools were not explicitly informed of their outcome, but intervention schools were aware of their allocation because the research officer worked closely with them to train and support them in the implementation toolkit. The study analyst and chief investigators remained blinded to allocation during the trial.

#### Procedure

This implementation-effectiveness trial was embedded into a pragmatic, large-scale roll out of the PAX Good Behavior Game across New South Wales, funded and coordinated by the State Department of Education. Schools were invited to the research study if they had registered ≥ 1 staff member to train in the PAX Good Behavior Game. The Department provided a list of schools to the research team, who emailed a study invitation to the principal that included a link to an online consent form.

Following receipt of the principal’s consent, schools were allocated to start the trial in either 2021 or 2022 (schools consented by end of February 2021 were in the first wave, all other schools were allocated to the second wave). Principals were sent a link to an individual-level participant information sheet and consent form that was unique to their school; they were asked to distribute this at the start of the year to any staff who were registered to train in the PAX Good Behavior Game by 31 March. Any staff who opted in to the research had to complete the study registration process (name; role in school — “classroom teacher” or “support staff”; contact details). This information was used to allocate them a unique trial identifier, determine what surveys they would be sent (both roles completed the implementation surveys; only classroom teachers completed the effectiveness survey), and establish their communication preferences.

### Data Collection

The timing for the initial (T0) implementation (adoption, acceptability, feasibility, appropriateness) and effectiveness (student emotional and behavioral problems) surveys was staggered. The effectiveness outcome was first assessed following registration (day 0), while implementation outcomes were not assessed until 42 days post-registration. This delay enabled participants with sufficient time to complete the PAX Good Behavior Game training and initiate program delivery so that early adoption could be assessed. The survey schedules converged at T1, with both the implementation and effectiveness outcomes re-assessed at 175 days post-registration, and at 310 days post-registration (T2). Participants had 14 days to complete each survey. Two reminder communications were sent via SMS and/or email before the survey link was deactivated. All trial procedures were conducted online, automated by Black Dog Institute’s web-based trial platform. No reimbursements were provided as per school policy.

### Interventions

#### PAX Good Behavior Game

Both the intervention and control groups received the PAX Good Behavior Game and had access to all the standard implementation supports developed by the program owners: mandatory training in the program, delivered virtually over one full day or two half-days by a master trainer from the US-based PAXIS Institute, physical materials needed for implementation (e.g., harmonica, posters, wrist bands), a comprehensive printed instructional manual, and access to online resources via www.paxis.org/my-resources/. The PAX Good Behavior Game is described in detail elsewhere (Johansson et al., 2020), but briefly, it is comprised of nine “micro” operant conditioning techniques (known as “evidence-based kernels”) that can be delivered individually, and at high frequency, throughout the school day. At least once per day, these kernels are delivered all together (thus creating the “Good Behavior Game”). During the “game,” students work in teams to stay on task for a prespecified time during a set period of classroom instruction. If teams do not contravene the classroom prosociality (relational) rules, they are collectively rewarded. The dosage of the strategies increases in frequency and duration over time (e.g., from once per week to several times per day) across the school year, and the rewards are increasingly delayed, activating the self-regulation process. The PAX Good Behavior Game is a modified version of the original program, to make it more aligned to trauma-informed and strengths-based approaches (Johansson et al., 2020).

In addition to the standard program supports outlined above, schools can train staff to become PAX partners, enabling them to coach peers and monitor fidelity of implementation; however, this particular add–on training was not available in NSW schools during this trial.

#### PAX Plus Implementation Toolkit

In addition to the PAX Good Behavior Game base implementation model, intervention schools received a manualized multicomponent implementation toolkit developed by researchers at the Black Dog Institute, Sydney, Australia. The toolkit was designed to enhance program delivery by overcoming common challenges Australians schools report in establishing new innovations, and to address barriers specific to the PAX Good Behavior Game that have been identified in the published international literature. We reviewed the existing literature on common delivery barriers for school-based interventions (Baffsky et al., 2023a) and mapped these to the Consolidated Framework for Implementation Research 2.0 (Damschroder et al., 2022). A list of 11 implementation strategies aligned to these barriers was compiled from the Expert Recommendations for Implementing Change (ERIC) project’s evidence-based compilation of School Implementation Strategies, Translating ERIC Resources (SISTER) (Cook et al., 2019). This pool was refined and reduced via an iterative consultation with more than 30 teachers, school leaders, and education department representatives from across New South Wales. A detailed description of the comprehensive PAX Plus design and development process is published elsewhere (Baffsky et al., 2023b). The final version of the PAX Plus toolkit included nine implementation strategies (Table 2). Six strategies support teacher motivation and self-efficacy: emailed reminders, a virtual peer learning network, performance feedback, and a recognition system. The remaining strategies included monthly researcher-led meetings with the school leadership team to troubleshoot challenges, guidance on appointing a program champion, and systems for monitoring fidelity. To support uptake, a research officer from the Black Dog Institute conducted an initial 30-min video call with the school principal (or an alternative designated program champion) to orient them to the toolkit, followed by monthly calls during the first 6 months of the active intervention period.

**Table 2:** Overview of component strategies in the PAX Plus implementation toolkit

*CFIR 2.0 determinant*	*ERIC SISTER strategy*	*PAX PLUS* *strategy*	*Dosage*
*High-level leaders — capability* *Culture — learning centeredness*	Inform local opinion leader and facilitation/problem-solving	Executive support to school leadership team and school champion through monthly online meetings	Monthly meetings for 6 months
*Implementation leads – opportunity; innovation deliverers — motivation and capability; culture — learning centeredness*	Identify and prepare program champions	Program champion: nominated as the central person for trouble shooting challenges and motivating other staff	Organizes events or activities at least once per month
*Innovation deliverers: motivation*	Alter and provide individual-level incentives	Recognition system for successful efforts to implement	At least once per month
*Innovation deliverers — opportunity and capability*	Remind school personnel	Reminders (and tips) for delivery of PAX GBG strategies	Use every day
*Partnerships and connections*	Promote network weaving	Peer learning network: Virtual Question and Answer forum	Accessed anytime; published weekly
*Access to knowledge and information* *Innovation: deliverers capability*	Conduct ongoing training	Provide micro-refresher training: PAX Chats (live, online)	Provided monthly
*Reflecting and Evaluating — innovation*	Audit and provide feedback	Audit and feedback: Guidance provided to school leaders for ways to do this	Undertake auditing monthly, discussed at monthly executive support meetings
*Access to knowledge and information* *Innovation: deliverers opportunity*	Develop educational resources	All-in-one list of resources (digital copy) to find key information, easily	Use as needed, staff sent electronic reminders about it once per month; recommend reminding staff of this resource at the first staff meeting each term

*PAX GBG*, PAX Good Behavior Game

### Measures

Our primary implementation outcome and all secondary outcome measures were assessed at all timepoints. Consistent with our trial design, the primary endpoint was program adoption. We developed a binary, bespoke item for adoption as there is currently no validated, standardized measure of this construct. Staff were asked at all survey timepoints whether they were currently using or supporting PAX Good Behavior Game (yes/no), consistent with the way “adoption” is typically defined in the broader implementation literature (i.e., as uptake or utilization of an innovation) (Proctor et al., 2023; Reilly et al., 2020).

The main secondary endpoint was intervention effectiveness, assessed as change in emotional and behavioral problems, and measured using the total difficulties score on the teacher-rated version of the 25-item Strengths and Difficulties Questionnaire (SDQ) (Goodman, 1997) for 4–10-year-olds. Teachers were asked to complete the SDQ for every student in their class, based on observation. Items are rated on a 3-point Likert scale from 0 (not true) to 2 (certainly true). A total difficulties score representing the severity of emotional and behavioral problems was generated by summing four “problem” sub-scales (comprising 20 items): emotional symptoms, conduct problems, hyperactivity/inattention, peer relationships problems. Total difficulties scores range from 0 to 40, and higher scores indicate more severe problems (Cronbach’s alpha 0.86). The five-item prosocial behavior sub-scale of the SDQ (Goodman, 1997) was used to measure change in behaviors that help or care for others. Prosocial behavior scores range from 0 to 10, with higher scores indicating more prosocial behavior (Cronbach’s alpha 0.79). The teacher-rated SDQ has demonstrated good construct validity across seven European countries (Husky et al., 2020).

The acceptability of implementation measure (AIM), feasibility of implementation measure (FIM), and intervention appropriateness measure (IAM) were used as secondary implementation outcome measures. Each of these is a four-item scale with total scores ranging from 4 to 20 (Weiner et al., 2017). Items are rated on a 5-point Likert scale from 1 (completely disagree) to 5 (completely agree). Higher scores indicate more favorable responses (Weiner et al., 2017). The AIM, FIM, and IAM have good construct validity and test–retest reliability (Cronbach *α* = 0.85, *α* = 0.89, *α* = 0.91, respectively) (Weiner et al., 2017).

At baseline, basic demographic characteristics of participants were captured via self-report. Information on school size, school location (urban/metro vs regional), and socio-educational advantage were captured using publicly available administrative data from the Australian curriculum, assessment, and reporting authority.

### Statistical Analysis

All analyses were undertaken on an intent-to-treat basis, including all participants as randomized, regardless of intervention received. Analyses included all available data from participants with at least one data point, with missing data assumed to be missing at random. The primary outcome analysis examined the effect of PAX Plus relative to control on changes in program adoption (response = yes/no) from T0 to T1. Program adoption at 12 months (T2) was not examined due to the issue of high attrition (73% missing data; see “Deviation from Study Protocol”). A generalized linear mixed model (GLMM) with a binomial distribution, a logit link, and maximum likelihood estimation was used for this analysis, with stratification variables (school size and regionality), time (T0, T1), group (intervention, control), and a time × group interaction specified as fixed effects. Random intercepts were specified for schools and staff (nested within schools) to reflect the clustered design and individual differences at T0, respectively. Effect sizes are presented as odds ratios (ORs).

Analyses of program effectiveness (secondary outcome) involved examining the effect of the PAX Plus intervention relative to control on changes in SDQ total difficulties scores and SDQ prosocial behavior scores from T0 to T1. Analyses of secondary implementation outcomes involved examining the effect of the PAX Plus intervention relative to control on changes in AIM, FIM, and IAM scores from T0 to T1. Linear mixed models (LMMs) with maximum likelihood estimation were used for these analyses, with the same fixed effects as previously specified for the GLMM, as well as random intercepts specified where appropriate for schools, staff (nested within schools), and students (nested within staff). The LMMs employed an identity covariance matrix, and degrees of freedom were estimated using Satterthwaite’s method. Effect sizes are presented as Cohen’s *d*, to reflect between- and within-group effects as appropriate, with calculations based on relevant modelled mean differences and raw SDs at relevant timepoints.

Any obtained significant differences at T0 between groups in primary outcomes, secondary effectiveness outcomes, or secondary implementation outcomes were further explored in post hoc GLMM or LMM analyses as appropriate which constrained the group modelled means at T0 to be equivalent (Coffman et al., 2016). This constraint allows group differences at T0 to be accounted for in the analysis.

A post hoc analysis was also conducted to explore whether gender moderated the effect of the PAX Plus intervention relative to control on changes in SDQ total difficulties scores. A LMM as previously described but with added terms for student gender and its interactions with time and group was used for this analysis.

Due to the nature of results, planned mediational analyses examining AIM, FIM, and IAM scores as potential mediators between the intervention and program adoption could not be conducted. Instead, a post hoc analysis examined whether changes in each of the AIM, FIM, and IAM scores were associated with change in program adoption. Bivariate LMMs with maximum likelihood estimation simultaneously modelled changes in an implementation outcome (AIM, FIM, or IAM scores) and program adoption, accounting for stratification variables and clustering at the school and staff levels as in previous models. Given the simultaneous modelling of outcomes, program adoption needed to be treated as a continuous variable in these analyses. The correlation between change in AIM, FIM, or IAM scores and change in program adoption, along with a bias-corrected and accelerated 95% confidence interval (BCa 95% CI), was estimated from 1000 bootstrap samples. The correlation was deemed significant if the BCa 95% CI did not contain 0.

For all analyses, statistical significance was set at *p* < 0.05. All data were analyzed using R version 4.4.1 and the R packages lme4 (Bates et al., 2015) and nlme (Pinheiro J et al., 2024).

### Deviation from Study Protocol

Our ANZCTR trial protocol specified that our primary outcome would be assessed at 12-month post-baseline. Due to significant COVID-related disruptions during the first recruitment wave, there were unanticipated delays in recruiting participants which contributed to an extremely high rate of attrition in both groups by the 12-month assessment (e.g., 73% missing data on adoption outcome). Accordingly, the 12-month assessment was abandoned for wave 2 recruitment, and the first wave 12-month data has not been included in this manuscript.

## Results

In total, 147 teachers and 40 support staff participated in either the baseline implementation and/or effectiveness survey (*n* = 130 teachers, 88.4% completed both surveys). Of these, 179 participants completed the primary outcome measure (adoption) at one or more timepoints (*n* = 171, 95.5% at T0; *n* = 110, 61.5% at T1). At T0, 90.5% (*n* = 133) of teachers completed the SDQ survey and 35.4% (*n* = 52) at T1 (Fig. 1). Demographic characteristics are presented in Table 1.

Control schools were not prohibited from establishing their own implementation supports, and participants in both conditions reported use of similar implementation strategies, although usage was greater in the intervention group (Table 3). The most commonly used strategies in both groups were reminders to use the PAX Good Behavior Game, peer learning opportunities, progress monitoring, and program champions. Rewards/incentives had little to no uptake.

**Table 3:** Implementation strategy usage, aligned to PAX Plus Toolkit

Implementation strategies	Intervention (*n* = 94)		Control (*n* = 93)	
	T0	T1	T0	T1
Identify and prepare program champions	58 (61.7)	33 (35.1)	20 (21.5)	10 (10.8)
Provide individual-level incentives (reward)	3 (3.2)	9 (9.6)	0	0
Progress monitoring/auditing	56 (59.6)	30 (31.9)	24 (25.8)	8 (8.6)
Remind school personnel (how) to use PAX GBG	77 (81.9)	36 (38.3)	38 (40.9)	9 (9.7)
Peer learning network and opportunities (*n*, %)	54 (57.4)	32 (34.0)	28 (30.1)	8 (8.6)
Refresher training	8 (8.5)	15 (16.0)	2 (2.2)	3 (3.2)
No. of strategies, *M* (SD)	3.18 (0.9)	3.95 (1.2)	2.02 (3.6)	3.17 (1.4)

*M*, mean; *SD*, standard deviation; *PAX GBG*, PAX Good Behavior Game

### Effect of Intervention on Program Adoption

The proportion of participants reporting program adoption at each timepoint is presented in Table 4, with high model-based rates of adoption in both groups at T1 (intervention 96.8%, control 95.7%). Initial model testing indicated model convergence issues. As such, a simpler model accounting only for clustering at the participant level was tested and found to converge. Adoption at T0 was higher in the intervention group (93.6%) compared to the control (45.2%) (OR = 21.20, 95% CI [3.50, 128.45], *z* = 3.32, *p* < 0.001). There was also a significant time × group interaction for adoption (*p* = 0.006; Table 4), reflecting a larger increase in adoption for the control group (OR = 36.19, 95% CI [6.09, 215.12], *z* = 3.95, *p* < 0.001) compared to the intervention group (OR = 2.06, 95% CI [0.55, 7.64], *z* = 1.08,* p* = 0.281). There was no between-groups difference in adoption at T1 (OR = 1.21, 95% CI [0.16, 9.04], *z* = 0.18, *p* = 0.86).

**Table 4:** Model-based, timepoint estimates for primary and secondary outcomes for the PAX Plus intervention and PAX GBG control groups

	Intervention		Control		Time			Group			Time × group		
	T0	T1	T0	T1	*OR* [95% CI]	Statistic	*p*	*OR* [95% CI]	Statistic	*p*	*OR* [95% CI]	statistic	*p*
Primary outcome													
Adoption, *n* (%)	88 (93.6)	91 (96.8)	42 (45.2)	89 (95.7)	8.63 [2.65, 28.13]	*z* = 3.58	< 0.001	4.60 [1.87, 11.33]	*z* = 3.32	< 0.001	0.24 [0.09, 0.67]	*z* = − 2.74	0.006
	T0	T1	T0	T1	*B* [95% CI]	statistic	*p*	*B* [95% CI]	statistic	*p*	*B* [95% CI]	statistic	*p*
Secondary outcomes													
SDQ total difficulties, *M* (*SE*)	8.31 (0.39)	7.07 (0.41)	9.41 (0.38)	7.53 (0.43)	− 1.56 [− 1.87, − 1.26]	*t* = − 10.06	< 0.001	− 0.55 [− 1.12, 0.03]	*t* = − 1.88	0.062	0.32 [0.01, 0.62]	*t* = 2.06	0.040
SDQ prosocial behavior, *M* (*SE*)	7.61 (0.13)	7.79 (0.14)	7.93 (0.13)	7.93 (0.15)	0.09 [− 0.01, 0.20]	*t* = 1.71	0.087	− 0.16 [− 0.36, 0.04]	*t* = − 1.59	0.115	0.09 [− 0.01, 0.20]	*t* = 1.69	0.092
AIM acceptability, *M* (*SE*)	16.18 (0.38)	17.55 (0.37)	15.33 (0.36)	16.20 (0.44)	1.12 [0.56, 1.67]	*t* = 3.98	< 0.001	0.43 [− 0.12, 0.97]	*t* = 1.55	0.124	0.25 [− 0.30, 0.80]	*t* = 0.88	0.379
FIM feasibility, *M* (*SE*)	16.07 (0.38)	17.00 (0.37)	14.81 (0.36)	15.16 (0.45)	0.63 [0.08, 1.18]	*t* = 2.27	0.025	0.63 [0.08, 1.18]	*t* = 2.25	0.025	0.29 [− 0.26, 0.84]	*t* = 1.05	0.296
IAM appropriateness, *M* (*SE*)	16.30 (0.37)	16.91 (0.36)	14.84 (0.35)	16.00 (0.44)	0.89 [0.28, 1.49]	*t* = 2.87	0.005	0.73 [0.20, 1.25]	*t* = 2.74	0.007	− 0.28 [− 0.88, 0.33]	*t* = − 0.90	0.372

Models in table included stratification variables (school size and regionality) as fixed effects

In a post hoc analysis which constrained adoption at T0 to be equivalent between groups, the time × group interaction was non-significant (OR = 0.41, 95% CI [0.15, 1.11], *z* = − 1.75, *p* = 0.08). The between-groups difference in adoption at T1 remained non-significant (OR = 0.41, 95% CI [0.15, 1.11], *z* = − 1.75,* p* = 0.08; note statistics are the same as the time × group interaction due to constraint that group means at T0 are equivalent). Adoption increased from T0 to T1 on average for both groups (OR = 25.81, 95% CI [4.38, 152.09], *z* = 3.59, *p* < 0.001).

### Effect of Intervention on Emotional and Behavioral Problems and Prosocial Behavior

#### Emotional and Behavioral Problems

SDQ total difficulties scores did not differ between groups at T0 (*B* = − 1.09, 95% CI − 2.25, 0.06],* t*[131.72] = − 1.88, *p* = 0.06, *d* = − 0.17). There was a time × group interaction for SDQ total difficulties scores (*p* = 0.04; Table 4), reflecting a significant decrease in the intervention group (*B* = − 1.24, 95% CI [− 1.62, − 0.87],* t*[977.03] = − 6.49, *p* < 0.001, *d* = − 0.20), but an even greater significant decrease in the control group (*B* = − 1.88, 95% CI [− 2.36, − 1.40], *t*[981.52] = − 7.70,* p* < 0.001, *d* = − 0.29). The overall T0 to T1 decrease in SDQ total difficulties scores across both groups was significant (*p* < 0.001, *d* = − 0.25; Table 4). There was no significant difference in SDQ total difficulties scores between groups at T1 (*B* = − 0.46, 95% CI [− 1.71, 0.80], *t*[184.19] = − 0.72, *p* = 0.47, *d* = − 0.08). In a post hoc analysis, student gender did not moderate the time × group interaction, nor the time or group effects of the previous analysis (see Table S1).

#### Prosocial Behavior

There was no difference between groups at T0 for SDQ prosocial behavior scores (*B* = − 0.32, 95% CI [− 0.72, 0.08], *t*[127.04] = − 1.59, *p* = 0.115, *d* = − 0.16), no time × group differences (*p* = 0.092; see Table 4), and no significant change in scores, on average, across the groups (*p* = 0.087, *d* = 0.04).

#### Effect of Intervention on Program Feasibility, Acceptability, and Appropriateness

At the implementation T0, there were no group differences for program acceptability scores (*B* = 0.86, 95% CI [− 0.24, 1.95], *t*[199.40] = 1.55, *p* = 0.12, *d* = 0.26), but feasibility scores (*B* = 1.26, 95% CI [0.16, 2.35], *t*[194.84] = 2.25, *p* = 0.025, *d* = 0.40) and appropriateness scores (*B* = 1.46, 95% CI [0.41, 2.51], *t*[215.36] = 2.74, *p* = 0.01, *d* = 0.48) were significantly higher in the intervention group. There were no differential time × group effects for acceptability (*p* = 0.38; Table 4), feasibility (*p* = 0.296), or appropriateness (*p* = 0.37). However, there were significant increases from T0 to T1 in acceptability (*p* < 0.001, *d* = 0.35; Table 4), feasibility (*p* = 0.03, *d* = 0.20), and appropriateness (*p* = 0.01, *d* = 0.29), on average, in both groups (Table 4).

In a post hoc analysis which constrained feasibility at T0 to be equivalent between groups, the time × group interaction was not significant (*B* = 0.49, 95% CI [− 0.02, 1.00], *t*[122.69] = 1.89, *p* = 0.062), there was no longer any significant change in program feasibility from T0 to T1 on average for the groups (*B* = 0.18, 95% CI [− 0.62, 0.98], *t*[117.95] = 0.45, *p* = 0.657, *d* = 0.06), and the time × group interaction for appropriateness was not significant (*B* = − 0.01, 95% CI [− 0.56, 0.54], *t*[133.94] = − 0.03, *p* = 0.98). However, the increase from T0 to T1 in appropriateness on average for the two groups remained significant (*B* = 0.94, 95% CI [0.06, 1.83], *t*[138.69] = 2.11, *p* = 0.04, *d* = 0.31).

### Associations Between Changes in Program Acceptability, Feasibility, and Appropriateness and Change in Program Adoption

As previous analyses showed increases in program adoption and increases in some of the implementation outcomes from T0 to T1 for both groups with no significant time × group effects, changes in each of the implementation outcomes and in program adoption were simultaneously modelled for the total sample from T0 to T1. Bivariate models showed significant increases in both program acceptability (*p* = 0.002; Table 5) and program adoption (*p* = 0.01). Program feasibility did not significantly change (*p* = 0.079) when jointly modelled with program adoption, which significantly increased from T0 to T1 (*p* = 0.01). Similarly, program appropriateness did not significantly change from T0 to T1 (*p* = 0.09) when jointly modelled with program adoption which significantly increased from T0 to T1 (*p* = 0.01). Nonetheless, increases in each of the implementation outcomes were significantly correlated with increases in program adoption (bootstrap samples *r* ranging from 0.23 to 0.31; Table 5).

**Table 5:** Changes in program acceptability, feasibility, and appropriateness and associations with change in program adoption

Implementation outcome	Change in implementation outcome				Change in program adoption				Correlation between changes (original sample)	Correlation between changes (bootstrap samples)
	*B* [95% CI]	statistic	*p*	*d*	*B* [95% CI]	statistic	*p*	*d*	*r*	*r* [BCa 95% CI]
AIM: acceptability	0.94 [0.34, 1.54]	*t* = 3.05	0.002	0.29	0.16 [0.05, 0.28]	*t* = 2.72	0.007	0.33	0.56	0.31 [0.13, 0.47]
FIM: feasibility	0.58 [− 0.06, 1.23]	*t* = 1.76	0.079	0.18	0.16 [0.04, 0.27]	*t* = 2.73	0.007	0.33	0.46	0.23 [0.05, 0.43]
IAM: appropriateness	0.63 [− 0.09, 1.34]	*t* = 1.72	0.086	0.20	0.17 [0.05, 0.28]	*t* = 2.80	0.005	0.34	0.56	0.28 [0.11, 0.43]

Models in table included stratification variables (school size and regionality) as fixed effects

*AIM*, acceptability of implementation measure; *FIM*, feasibility of implementation measure; *IAM*, intervention appropriateness measure; *BCa 95% CI*, bias-corrected and accelerated 95% confidence interval

## Discussion

This hybrid implementation-effectiveness cluster RCT found no effects of a multicomponent implementation strategy on the adoption or effectiveness of the PAX Good Behavior Game, compared to implementation as usual. The rate of early adoption was twofold higher in the intervention group at 6 weeks; however, these differences had disappeared by 6 months, with both groups reporting rates of program adoption of > 90%. A nearly identical pattern of program uptake was observed in a US implementation science trial, testing if an adaptive behavior change training strategy increased adoption of the GBG program over a similar time period (Merle et al., 2023). Perceived program appropriateness, feasibility, and acceptability were positively, linearly associated with program adoption in both groups, suggesting the program is contextually relevant and feasible for Australian primary schools. There were small, significant improvements in student emotional and behavioral problems over time in both groups, with effect sizes in line with prior standard superiority trials of the Good Behavior Game (Kellam et al., 2011). Our findings support the effectiveness of the standard (base) implementation model for the PAX Good Behavior Game, and that our additional implementation supports may help speed up the adoption process but do not optimize implementation or effectiveness over time.

### Effect of Implementation Strategies on Adoption

There are several potential explanations for our adoption findings. The control group reported some organic use of implementation strategies analogous to those included in the PAX Plus toolkit, but at a lesser rate than the intervention group, likely due to their lack of access to a manualized toolkit and centralized research support. It is not surprising, however, that educators in control schools showed a strong willingness to problem-solve for implementation. The trial took place during the COVID-19 pandemic, which created the conditions for a remarkable deterioration in the mental health of children, globally (Hossain et al., 2022). Educators were likely highly motivated to implement initiatives to counter these effects and were funded to do so by the State Department of Education. The similarities in implementation strategies across both groups are consistent with the idea that the toolkit, which was iteratively developed with educators (Baffsky et al., 2023b), is a compendium of the experiential knowledge of educators in overcoming common barriers to implementation in Australian schools. The convergence in the levels of adoption at 6 months across groups is indicative of the high “implementability” of the standard version of the PAX Good Behavior Game, rather than the efficacy of PAX Plus, given the decline in the use of the implementation supports over time in both groups. Similar to those reported in Merle et al. (2023), our findings indicate that there is high uptake of implementation supports in the establishment phase of a new program that are consistent with building teachers’ confidence and motivation to deliver (i.e., school champions, reminders, training, and peer-support), but their relevancy diminishes as self-efficacy is established.

#### Effect of Implementation Strategies on Effectiveness Outcomes

Our trial showed a small, significant, but potentially important, reduction in emotional and behavioral problems among students in both groups. The magnitude of the effect for this change was comparable to measures of similar behavioral problems in previous trials of the Good Behavior Game (*d* = − 0.25 versus *g* = 0.010–0.49, respectively), indicating that the toolkit did not optimize effectiveness as hypothesized. When the reductions in emotional and behavioral problems are considered alongside the high adoption rates in both groups, the hypothesis that good implementation moderates effectiveness appears to be supported. There was no effect on prosocial behaviors despite prosociality being a core focus of the PAX program. This null finding may be an artifact of measurement biases rather than a true intervention effect. The prosocial items measured in the SDQ (e.g., frequency of volunteering, kindness to junior peers, helping others in pain) do not accurately reflect how prosociality is conditioned in the PAX Good Behavior Game, where the focus is on promoting behaviors that support learning and cooperation in the classroom (Johansson et al., 2020). Concerns about the sensitivity of the SDQ to detect change in prosocial behaviors over time have been documented elsewhere (Kankaanpää et al., 2023).

#### Strengths and Limitations

The study had several strengths. To our knowledge, it is the first hybrid design randomized controlled implementation science trial conducted in Australia to test the effects of an evidence-informed, co-iterated implementation strategy, with the largest sample in this area, globally (*n* = 25 schools, 187 educators). Previously published implementation studies have been limited by the lack of rigorous experimental design and small sample sizes that are less than half of our study (e.g., < 10 schools, < 90 participants) (Baffsky et al., 2023a).

The findings, however, are also subject to important limitations. We did not measure certain elements of fidelity, including dosage and frequency of the PAX Plus toolkit that could help contextualize adherence to the implementation protocol. Toolkit usage data was only measured using teacher self-report, which could not be validated due to restrictions on entering schools due to COVID-19 pandemic, and may be subject to several significant self-report biases. Therefore, the quality of implementation adherence in this study is unknown. Between 39% and 65% of participants had missing data on key variables of interest at T1, which was higher than anticipated in our a priori calculations, likely limiting power to detect effects in the primary outcome. The trial occurred during the height of the COVID-19 pandemic, causing high rates of distress and burnout in the teaching workforce, which may have contributed to this high attrition. There was a potential sampling bias as only 28 out of 70 invited schools (40%) agreed to take part in the trial. Our sample could be overly representative of schools that value evidence-based practices, accounting for the high rates of adoption, and limiting the generalizability of our results. While allocation at the cluster level resulted in a balanced school level sample, our reliance on principals to distribute study invites within schools meant we were unable to ensure balance at the participant level. There were baseline imbalances that may have influenced outcomes, i.e., overrepresentation of staff from small schools and regional areas in the intervention group. Finally, effectiveness was assessed using only teacher-report. There is some evidence that teachers overestimate externalizing problems in the SDQ (Goodman et al., 2000), potentially obfuscating study outcomes.

#### Future Research

This study provides some answers to “if” a multi-strategy implementation approach works but leaves many questions unaddressed as to how it worked, whom it worked for, in what contexts it works, and whether a multi-strategy approach is any more effective than a single strategy approach. Future research should consider using dismantling trials with longitudinal evaluations to examine what specific implementation strategies drive intervention response, by what mechanisms they produce change, and when they exert the most influence, to enable the development of more precise, resource-efficient implementation strategies across the program lifecycle. Identifying contextual factors that explain variance in program outcomes will help us better understand the conditions for effectiveness and which schools benefit most from implementation support, enabling more focused interventions in the future. Low-quality implementation can obscure true intervention effects. Future research should develop and validate scalable fidelity assessment methods, such as technology-enhanced observation or analytics-based monitoring, to enable reliable, efficient measurement of implementation quality, particularly in large-scale trials, and address potential confounding. As insufficient training is a necessary foundation for self-efficacy and high-quality delivery (Sadjadi et al., 2022), researchers should investigate if, and how, new technologies like generative AI can effectively and efficiently build workforce capacity relative to traditional training methods.

Overall, our findings show that the PAX Good Behavior Game is likely to be an effective, highly acceptable, and feasible prevention approach for addressing early emotional and behavioral dysfunction indicators of mental health disorders. On this basis, we recommend that education departments invest in building local capacity to deliver the program, embed it into statewide and national well-being and school improvement policies as an example of evidence-based practice, and promote awareness of it through information directories that schools already use. Aligned to this, a monitoring and surveillance evaluation framework should be established to understand its ongoing impacts, to ensure it remains responsive to the needs of staff and students, and to enable evidence-based decisions regarding its sustainability. Although the program is highly implementable, teachers still appeared to benefit from the provision of additional implementation supports related to building self-efficacy in, and motivation for, program delivery. However, the cost–benefits of ongoing use of, and investment in, implementation supports are unclear. Future studies need to identify what strategies teachers may benefit from, at what point, to achieve high-quality, efficient, and sustainable implementation.

## Supplementary Information

Below is the link to the electronic supplementary material.

ESM 1 (DOCX 17.3 KB)

## Data Availability

Participant data that underlie the results reported in this Article, after deidentification (text, tables, figures, and appendices), may be shared by at an aggregated level by the corresponding author on reasonable request for academic and research purposes.

## Declarations

### Trial Registration

The protocol was retrospectively registered with the Australian New Zealand Clinical Trials Registry, ACTRN12621001125819.
