# Supplementary material for: Using a Multicomponent Implementation Strategy to Increase Adoption and Effectiveness of a Universal Mental Health Prevention Program in Australian Primary Schools: a Cluster Randomized Trial Using a Type-3 Hybrid Design
Source: Prev Sci. 2026 Feb 6;27(2):301–15. doi: 10.1007/s11121-025-01870-3 (PMC12999845; doi:10.1007/s11121-025-01870-3)
Supplement: Supplementary file 1 — (DOCX 17.3 KB) [file 11121_2025_1870_MOESM1_ESM.docx]

**Table S1. Examination of student gender as a moderator of the effect of the PAX Plus Intervention condition and the PAX GBG Control condition on SDQ total difficulties scores**

|  | *B* [95% CI] | *t* | *df* | *P* |
| --- | --- | --- | --- | --- |
| School type | -1.40 [-2.38, -0.42] | -2.82 | 124.82 | .006 |
| School size | -1.57 [-2.68, -0.46] | -2.81 | 129.26 | .006 |
| Gender | -2.72 [-3.12, -2.32] | -13.33 | 3213.47 | < .001 |
| Time | -1.70 [-2.11, -1.29] | -8.09 | 1071.65 | < .001 |
| Condition | -0.68 [-1.27, -0.09] | -2.27 | 163.64 | .024 |
| Gender x Time | 0.27 [-0.31, 0.85] | 0.90 | 1193.30 | .367 |
| Gender x Condition | 0.21 [-0.19, 0.61] | 1.04 | 3214.70 | .300 |
| Time x Condition | 0.09 [-0.33, 0.50] | 0.41 | 1071.98 | .678 |
| Gender x Time x Condition | 0.48 [-0.10, 1.06] | 1.61 | 1193.18 | .107 |
